# Kinetic characterization of lactate dehydrogenase in normal and malignant human breast tissues

**DOI:** 10.1186/s12935-015-0171-7

**Published:** 2015-02-15

**Authors:** Abdolhassan Talaiezadeh, Ali Shahriari, Mohammad Reza Tabandeh, Payam Fathizadeh, Siavash Mansouri

**Affiliations:** Cancer, Petroleum and Environmental Pollutants Research Center, Ahvaz Jundishapur University of Medical Sciences, Ahvaz, Iran; Department of Biochemistry and Molecular Biology, Faculty of Veterinary Medicine, Shahid Chamran University of Ahvaz, Ahvaz, Iran

**Keywords:** Breast cancer, Aerobic glycolysis, Lactate dehydrogenase, Enzyme kinetic

## Abstract

**Background:**

Aerobic glycolysis rate is higher in breast cancer tissues than adjacent normal tissues which providethe ATP, lactate and anabolic precursors required for tumourgenesis and metastasis. Lactate dehydrogenase (LDH) is a critical enzyme during aerobic glycolysis as it is typically responsible for the production of lactate and regeneration of NAD^+^, which allows for the continued functioning of glycolysis even in the absence of oxygen. LDH has been found to be highly expressed in breast tumors. Enzyme kinetic characteristics is related to environmentinvolving the enzyme, and tumor microenvironment has distinct features relative to adjacent normal tissues, thus we hypothesized that LDH should have different kinetic characteristics in breast tumors compared to normal breast tissues.

**Methods:**

LDH was partially purifiedfrom human breast tumors and normal tissues, which were obtained directly from operating room. TheMichaelis-Menten constant (K_m_), maximum velocity (V_max_), activation energy (E_a_) and enzyme efficiency in breast tumors and normal tissueswere determined.

**Results:**

It was found that tumor LDH affinity in forward reaction was the same as normal LDH but V_max_ of cancerous LDH was higher relative to normal LDH. In reverse reaction, affinity of tumor LDH for lactate and NAD^+^ was lower than normal LDH, also enzyme efficiency for lactate and NAD^+^ was higher in normal samples. The E_a_ of reverse reaction was higher in cancerous tissues.

**Conclusions:**

It was concluded that thelow LDH affinity for lactate and NAD^+^ is a valuable tool for preserving lactate by cancer cells. We also conclude that increasing of LDH affinity may be a valid molecular target to abolish lactate dependent tumor growth and kinetic characteristics of LDH could be a novel diagnostic parameter for human breast cancer.

## Background

Excessive growth is an important characteristic of cancer cells. One of the main distinguishing features between the normalcells and cancer cells is in their intermediary metabolism [1]. Glycolysis and oxidative phosphorylation are two major metabolism pathways for producing ATP in mammalian cells [2]. Although oxidative phosphorylation produces higher ATP from one mole of glucose when compared to glycolysis, many questions remain about the efficiency of these pathways for support of excessive growth in cancer cells. According to the basic economic law of supply and demand, oxidative phosphorylation in normal cells is more efficient than glycolysis, but this does not apply to cancer cells. Cancer cells mainly generate ATP through glycolysis even in the presence of normal oxygen pressure [3]. Conversion of glucose to lactic acid in the presence of oxygen is known as aerobic glycolysis or the Warburg effect. Increased glycolysis is mostly observed in cancer cells. This bioenergetics and metabolic feature not only permits cancer cells to survive under adverse conditions such as hypoxia, but also enables their proliferation, invasion and subsequent distant metastasis. This condition alters cellular microenvironment and makes it toxic for other cells, but has no harmful effect on cancer cells [4]. High glycolysis results in environmental acidosis that facilitates invasion of cancer cells through destruction of adjacent normal populations, degradation of the extracellular matrix and promotion of angiogenesis [5].

The inhibition of Warburg effect may be used to attenuate the growth advantages of cancer cells; however, its precise molecular mechanisms are not completely understood. Different mechanisms have been described for glycolysis alteration in cancer cells [6,7]. One possiblereason for this bioenergetics alteration is the release of various enzyme activators or inhibitors, which can change the kinetic properties of enzymes involved in glucose metabolism. Environmental parameters like pH, temperature, or nutrient availability can influence enzyme activities and characteristics via transcriptional, posttranscriptional, posttranslational, or allosteric regulations. Slight attention has been paid to alteration of enzyme kinetic in tumor environment, which can change intrinsic characteristics of enzymes and metabolic pathways.

LDH is the final enzyme in glycolysis pathway that catalyzes interconversion of pyruvate and lactate and it also regenerates NAD^+^, which isnecessary for continued high glycolysis rate in cancer cells. The gene expression and activity of LDH (in pyruvate reducing direction) is higher in breast cancer cells relative to adjacent normal cells. Also, upregulation of the LDH-A in clinical tumors is often associated with disease progression and poor prognosis [8-12]. Interestingly, clinical evaluation of LDH-B could be a predictive marker of response for patients with breast cancer receiving neoadjuvant chemotherapy [13]. Given the findings, it is concluded that LDH is animportant effector of glucose metabolism in cancer cells and can affect tumorigenesis and metastasis. Possible changes in kinetic parameters of LDH with attention to different tumor microenvironmentshave not been studied in cancer tissue. The aim of the current study was to compare the kinetic properties of lactate dehydrogenase between breast cancer and normal mammary tissue.

## Material and methods

### Clinical sample collection

Seventeen human breast tumor samples were obtained from Apadana Hospital during the surgery. Normal tissues away from the tumor were included as controls. Two independent expert pathologists from the pathology laboratory of Apadana hospital carried out the pathological tumor and control tissue examination. The clinical and histological characteristics of breast cancer patients are shown in Table 1. Samples were immediately preserved in liquid nitrogen, transported to the laboratory, and stored at −80°C. The study was approved by the ethics committee from Jundishapour Medical University of Ahvaz and conducted according to the Guide for Human study by the National Academy of Sciences (National Institutes of Health), and informed consent was obtained from all patients involved in this study.

**Table 1:** **Clinico-pathological characteristics of the examined breast cancer patients**

**Clinical characteristics**	**Grade**	**% of patients**
Age (years)	<40	35.29
	40–49	23.52
	50–59	35.29
	60–69	5.8
Histopathological grade	1 grade	14.28
	2 grade	57.14
	3 grade	28.57
Cellular characteristics	Ductal carcinoma invasive	86.66
	Ductal carcinoma in situ	13.33
Tumor size	2.1–3.0	13.33
	>3.1	86.66
Auxillary lymph nodes status	Metastasis negative	33.33
	Metastasis positive	66.66

### Sample preparation and LDH partial purification

Frozen tumors and normal tissues were homogenized (1:5 w:v) in ice cold homogenization buffer (20 mMTris–HCl, pH 8.0, 10 mM 2-mercaptoethanol, 10% v:v glycerol, 2 mM EDTA, 2 mM EGTA, and 20 mM β-glycerophosphate) and a few crystals of phenylmethylsulphonyl fluoride (PMSF) were added at the time of homogenization. Samples were homogenized using a Miccra homogenizer (Miccra, Germany), centrifuged for 30 min at 13,500 g at 4°C and the supernatant was decanted and held on ice until use. Low molecular weight metabolites and ions were removed from the supernatant by Sephadex G25 columns (1 × 5 cm) (Sigma, Germany) that had been equilibrated in homogenizing buffer [14].

LDH partial purification began with the preparation of a DEAE-Sephasex column (1.5 × 10 cm) that was equilibrated in assay buffer (20 mM Tris–HCl, pH 8). Following equilibration, approximately 1.5 mL of crude extract was placed on top of the column. The column was then washed with 30 mL of assay buffer to remove any unbound proteins like LDH.

Additional experiments required a much pure LDH sample, and therefore the top peak activity fractions from the DEAE-Sephadex column were combined and chromatographed on a Blue Sepharose CL-6B column (1.5 × 10 cm) pre-equilibrated in homogenization buffer. Following equilibration, the column was then washed with 50 mL of homogenization buffer to remove any unbound proteins. A linear salt gradient of 0–2 M KCl was then applied to the column for the elution of LDH. Top activity fractions were then pooled and held at 4°C until use. This sample was used for subsequent kinetic characterization of LDH [14].

### Enzyme assay and kinetic parameters

LDH activity was measured in the presence of pyruvate with NADH as substrate for forward reaction and lactate with NAD^+^ as substrate for reverse reaction. The lowest saturating concentration of each substrate, which simultaneously showed maximum velocity, constant rate of product formation and linear regressions of activities for serial dilutions of enzyme, was assigned as optimum substrate concentration.

Reactions were initiated by adding 10 μl of purified enzyme to a 200 μl total reaction volume by using 20 mM Tris–HCl buffer pH 8, in the microplate well. Activity was monitored at 340 nm for checking the conversion of NADH to NAD^+^ (or vice versa) by using a Biotech Powerwave X2s microplate reader (Biotech, USA) and Gen5 software version 2.0 (USA) (kinetic mode, reading interval = 39 s). The enzyme activity was expressed as nmoles of pyruvate or lactate formed/min for reverse and forward reactions, respectively.

Data were analyzed using microplate analysis (MPA) and kinetics programs 3.51 [15,16]. Kinetics 3.51 computer program fitted data through a nonlinear least squares regression for determination of K_m_ (substrate concentration giving half-maximal activity; Michaelis-Menten constant) and V_max_ (maximum velocity) values.

The K_m_ pyruvate was determined at 0.5 mM NADH and pyruvate concentrations ranged from 0.05 mM to 2.15 mM. The K_m_ NADH was determined at 1.5 mM pyruvate and NADH concentrations ranged from 0.1 to 0.95 mM. In normal tissues, the K_m_ lactate was determined at 3 mM NAD^+^ and lactate concentrations ranged from 5–115 mM, while the K_m_ NAD^+^ was determined at 90 mM lactate and NAD^+^ concentrations ranged from 0.25 to 8.5 mM. In tumor samples, the K_m_ lactate was determined at 5 mM NAD^+^ and lactate concentrations ranged from 10–325 mM while the Km NAD^+^ was determined at 250 mM lactate and NAD^+^ concentrations ranged from 0.5 to 9.5 mM. All assayswererun in 0.2 M Tris-HCl buffer, pH 8.0. All reactions were done in triplicate. The K_m_ and V_max_ were calculated from the mean of three separate series of determinations. Total protein content was measured using Bradford method and bovine serum albumin as standard.

Given the possible existence of endogenous NADH to NAD^+^ interconvesrion (i.e. NADH oxidation by complex І activity) in partial purified samples, the NADH to NAD^+^interconversion was surveyed in each sample to eliminate the possible existence of its effect. This interconversion was determined by adding the NADH (0.5-1 mM) or NAD^+^ (3–5 mM) in Blue Sepharose purified samples and monitoring the change in absorbance at 340 nm.

### Calculation of activation energy

Maximal LDH activity was determined at 5°C increments starting from 18°C and ending at 42°C. The reaction temperature was set by using incubator of Biotech Powerwave X2s microplate reader. Substrates concentrations were as follow: 1.5 mM pyruvate and 0.5 mM NADH in forward reaction, 90 mM lactate and 3 mM NAD^+^ in reverse reaction for normal tissues, 250 mM lactate and 5 mM NAD^+^ in reverse reaction for tumor tissues. Arrhenius plots were constructed from these experiments and the E_a_ was calculated.

### Calculation of enzyme efficiency

To determine enzyme efficiency the V_max_/K_m_ lactate and V_max_/K_m_ NAD^+^ ratio were calculated for tumor and normal samples.

### Statistical analysis

Data were expressed as mean ± SEM from independent determinations on separate preparations of enzyme. Data were analyzed using Student’s *t*-test. The level of significance for all tests was set at *p* < 0.05.

## Results

### LDH partial purification

**Figure 1:**
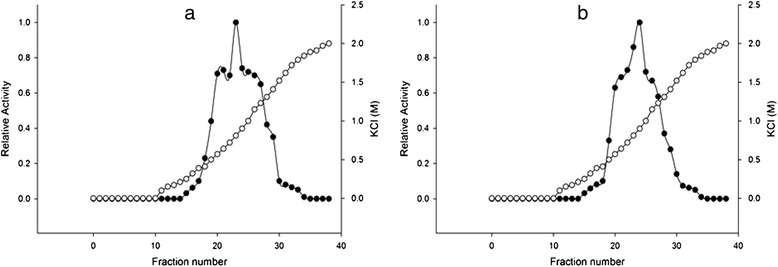
**Blue Sepharose CL-6B elution profiles for LDH activity from tumor and normal human breast tissues.** Activities are expressed relative to the highest activity fraction. **(a)** and **(b)** LDH elution profiles from tumor and normal breast top fractions of DEAE–G50 Sephadex on Blue Sepharose CL-6B, respectively. ●, LDH activity; ○, KCl concentration.

The purification procedure employed was proven to be efficient; a typical purification experiment is summarized in Tables 2 and 3. DEAE–G50 Sephadex chromatography resolved one peak activity of LDH from tumor and normal breast samples and Blue Sepharose chromatography determined one peak activity of LDH from top fractions of DEAE-G50 chromatography from both the tumor and normal samples. Peak activity of LDH was consistently eluted in Blue Sepharose at 0.5-1.3 M KCl from both tumor and normal breast specimens. The elution patterns of LDH showed no significant difference between tumor and normal breast tissues (Figure 1).

**Table 2:** **Purification scheme for LDH in cancerous breast samples**

**Purification step**	**Total protein (mg)**	**Total activity (U)**	**Specific activity (U/mg)**	**Fold purification**	**% yield**
Supernatant	15	5.1	0.3	-	-
DEAE-Sephadex	3.6	2.7	0.7	2.3	52
Blue Sepharose CL-6B	0.2	0.73	3.6	12	14

**Table 3:** **Purification scheme for LDH in normal breast samples**

**Purification step**	**Total protein (mg)**	**Total activity (U)**	**Specific activity (U/mg)**	**Fold purification**	**% yield**
Supernatant	8.5	2.4	0.28	-	-
DEAE-Sephadex	1.9	1.35	0.71	2.5	56
Blue Sepharose CL-6B	0.18	0.42	2.3	8.2	17

### Optimization of experimental conditions

Optimum assay conditions for LDH in forward reaction were 1.5 mM pyruvate and 0.5 mM NADH in both tumors and normal tissues. In the reverse reaction, optimal conditions were different in normal and tumor samples. Optimum conditions in normal samples were 90 mM lactate and 3 mM NAD^+^ while in tumor samples theywere 250 mM lactate and 5 mM NAD^+^. It should be noted that there is no NADH to NAD^+^ interconversion activity (or vice versa) in partially purified samples.

### Kinetic properties of LDH in forward reaction

**Figure 2:**
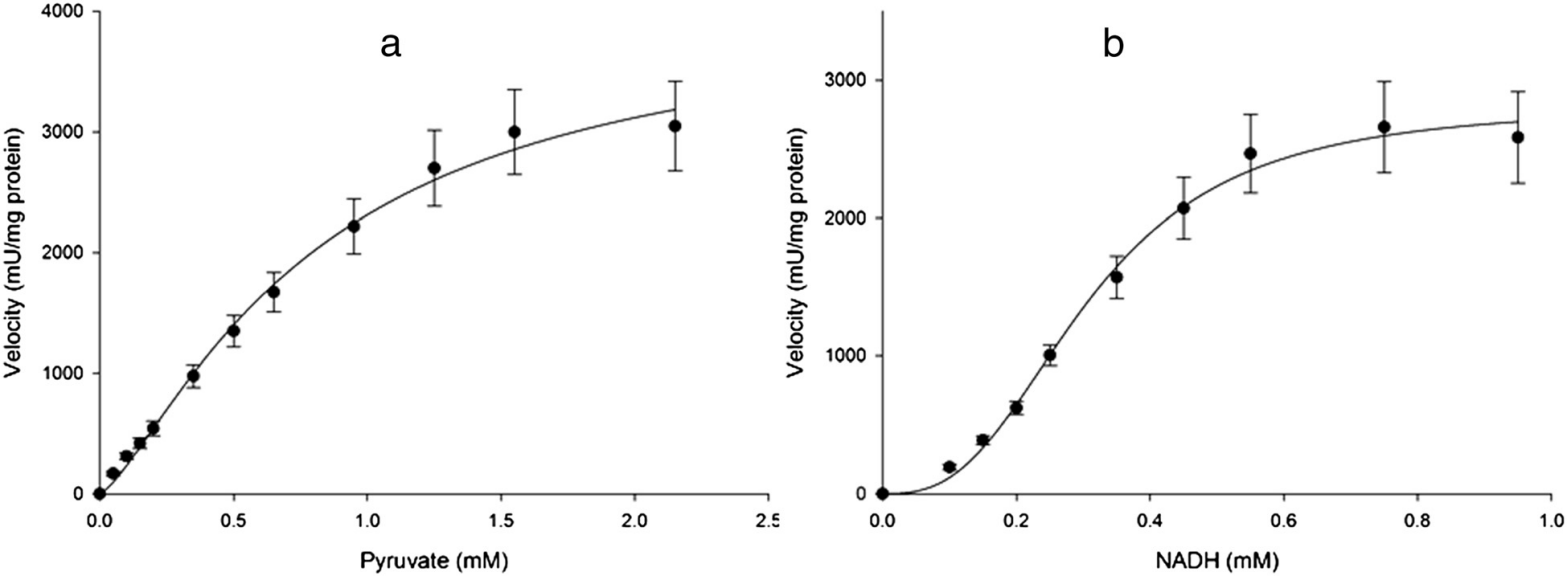
**Initial velocity (vi) versus substrate concentration of (a) Pyruvate: 0.05-2.15 mM and (b) NADH: 0.1-0.95 mM for LDH in partial purified of breast cancer samples (n =17) in forward reaction.** Data are presented as means ± SEM, n = 3 independent determinations on separate enzyme samples.

**Figure 3:**
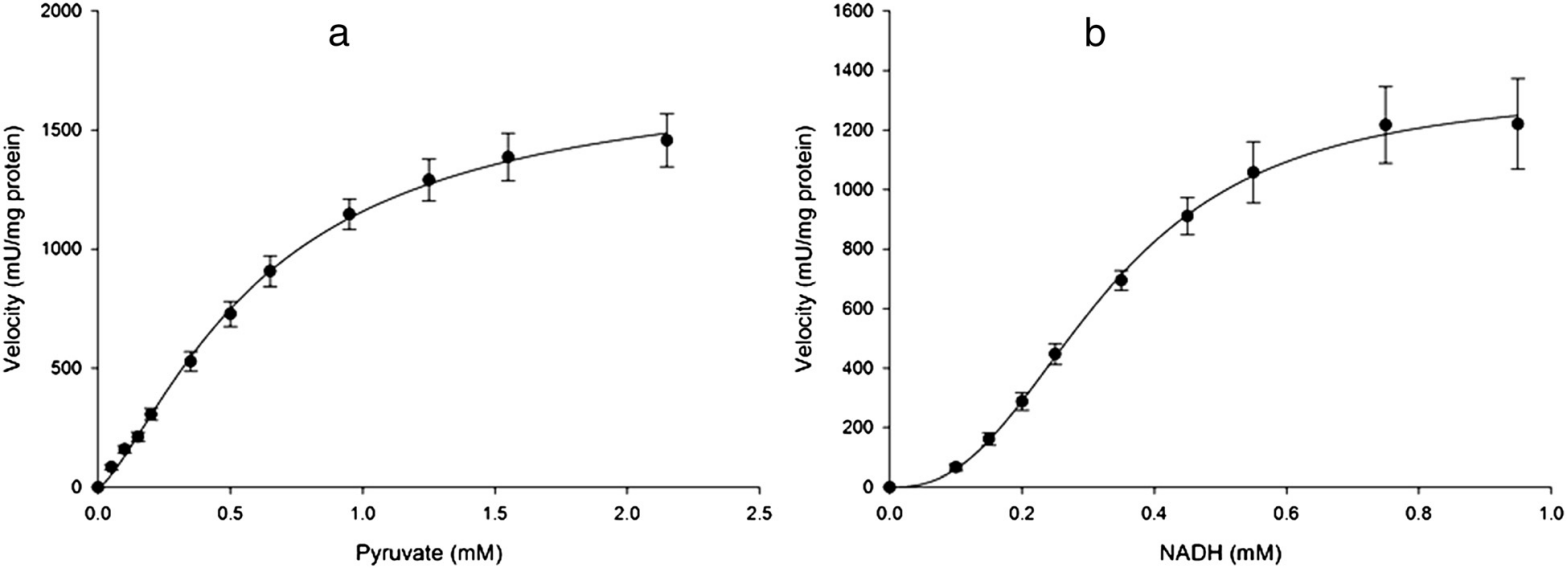
**Initial velocity (vi) versus substrate concentration of (a) Pyruvate: 0.05-2.15 mM and (b) NADH: 0.1-0.95 mM for LDH in partial purified breast normal tissues (n =17) in forward reaction.** Data are presented as means ± SEM, n = 3 independent determinations on separate enzyme samples.

The maximal activity of cancerous-LDH (C-LDH) for lactate formation (4034 ± 348 mU/mg protein for pyruvate and 2788 ± 111 mU/mg protein for NADH) was higher than the values in normal tissues (N-LDH) (1747 ± 68 mU/mg protein for pyruvate and1370 ± 52 mU/mg protein for NADH) (Table 4). The enzyme in forward reaction in both tissues displayed sigmoidal kinetics with respect to pyruvate and NADH (Figures 2 and 3). Hill coefficients for pyruvate and NADH in tumors were 1.36 ± 0.12 and 2.8 ± 0.28, and in normal samples were 1.36 ± 0.07 and 2.5 ± 0.19 respectively. However, the S_0.5_ of pyruvate (0.78 ± 0.12 mM for tumor and 0.63 ± 0.04 mM for normal) and NADH (0.3 ± 0.01 mM for tumor and 0.33 ± 0.01 mM for normal) were not significantly different between normal and cancerous tissues (Table 4).

**Table 4:** **Kinetic parameters of LDH in forward reaction from breast tumors (n =17) and normal tissues (n = 17)**

	**Tumor**	**Normal**
S _0.5_ pyruvate (mM)	0.78 ± 0.07	0.63 ± 0.03
S _0.5_ NADH (mM)	0.3 ± 0.01	0.33 ± 0.01
V_max_ pyruvate (mU/mg protein)	4034 ± 348*	1747 ± 68
V_max_ NADH (mU/mg protein)	2788 ± 111*	1370 ± 52
E_a_ (kcal/mol)	51 ± 4.1	41 ± 5.6

Assays were conducted at 25°C and data are presented as means ± SEM, n = 3 independent determinations on each of 17 tumor and normal samples.*Significant difference in each row at p < 0.05.

### Kinetic properties of LDH in reverse reaction

The maximal activity of LDH with respect to lactate (630 ± 4.9 mU/mg protein for tumor and 602 ± 2.2 mU/mg protein for normal) and NAD^+^ (1282 ± 71.24 mU/mg protein for tumor and 1237 ± 21.2 mU/mg protein for normal) were not significantly different between normal and cancerous tissues (Table 5).

**Table 5:** **Kinetic parameters of LDH in reverse reaction from breast tumors (n = 17) and normal tissues (n = 17)**

	**Tumor**	**Normal**
K_m_ lactate (mM)	21.78 ± 1.07*	10.73 ± 0.54
K_m_ NAD^+^ (mM)	0.99 ± 0.05*	0.50 ± 0.06
V_max_ lactate (mU/mg protein)	630 ± 4.9*	602.2 ± 2.2
V_max_ NAD^+^ (mU/mg protein)	1282 ± 71.24*	1237 ± 21.2
E_a_ (kcal/mol)	39.12 ± 4.6*	16.78 ± 1.7

Assays were conducted at 25°C and data are presented as means ± SEM, n = 3 independent determinations on each of 17 tumor and normal samples. *Significant difference in each row at p < 0.05.

**Figure 4:**
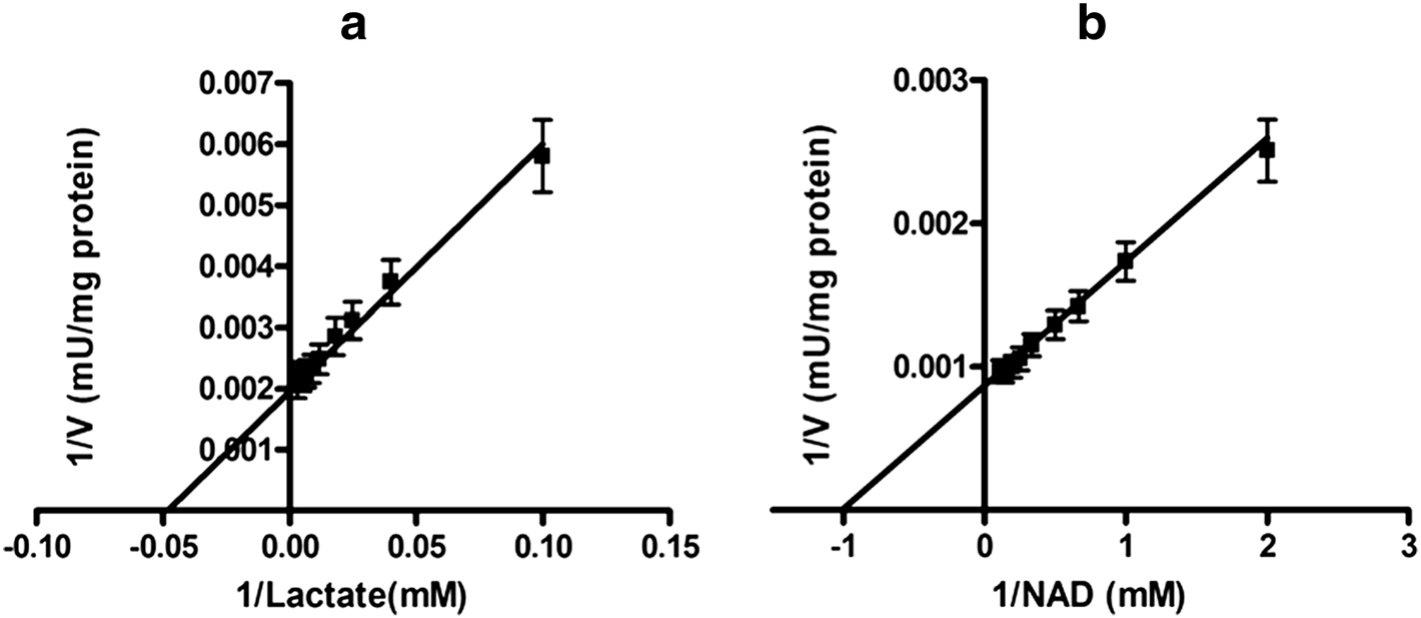
**Lineweaver-Burk plots of LDH in partial purified breast cancer tissues (n =17) for (a) Lactate: 10–325 mM and (b) NAD** ^**+**^ **: 0.5-9.5 mM in reverse reaction.** Data are presented as means ± SEM, n = 3 independent determinations on separate enzyme samples.

**Figure 5:**
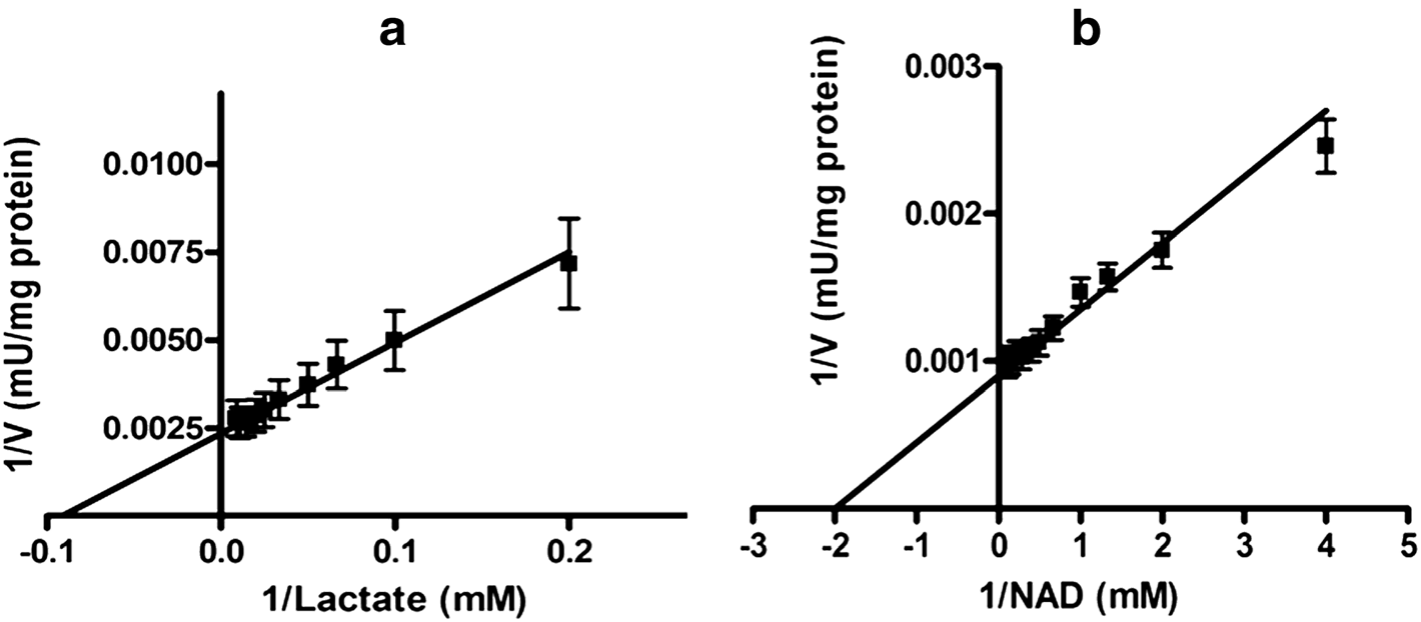
**Lineweaver-Burk plots of LDH in partial purified breast normal tissues (n =17) for (a) Lactate: 5–110 mM and (b) NAD** ^**+**^ **: 0.25-8.5 mM in reverse reactions.** Data are presented as means ± SEM, n = 3 independent determinations on separate enzyme samples.

The K_m_ lactate of N-LDH (10.73 ± 0.54 mM) was significantly lower than that of C-LDH (21.78 ± 1.07 mM) (p < 0.05) (Table 4). The K_m_ NAD^+^ was significantly lower in normal tissues (0.5 ± 0.06 mM) than that in tumor tissues (0.99 ± 0.2 mM) (p < 0.05) (Figures 4 and 5).

### Effect of temperature on LDH activity

E_a_ values of C-LDHL and N-LDH are shown in Table 4 and 5. The E_a_ in forward reaction was not significantly different between thetwo tissues (51 ± 4.1 Kcal/mol for tumor and 41 ± 5.6 Kcal/mol for normal tissue), while the E_a_ in reverse reaction was significantly higher in cancerous tissues (39.12 ± 4.6 Kcal/mol) compared to that in normal tissues (16.78 ± 1.7 Kcal/mol) (p < 0.05).

### Enzyme efficiency

Enzyme efficiency related to lactate in normal tissues was two-fold higher than tumor samples (56.1 ± 2.4 and 28.92 ± 1.3 mU/mg/mM, respectively). In the case of NAD^+^, enzyme efficiency in normal tissues was about two-fold higher than tumor samples (2474 ± 8.1 and 1294.94 ± 6.7 mU/mg/mM, respectively) (Table 6).

**Table 6:** **The enzyme efficiency, represented as V**_**max**_ **/K**_**m**_ **for LDH in partial purified tumor and normal breast tissues**

	**Tumor**	**Normal**
V_max_/K_m_ with lactate (mU/mg/mM)	28.92 ± 1.3*	56.1 ± 2.4
V_max_/K_m_ with NAD^+^ (mU/mg/mM)	1294.94 ± 6.7*	2474 ± 8.1

Assays were conducted at 25°C and data are presented as means ± SEM, n = 3 independent determinations on each of 17 tumor and normal samples. *Significant difference in each row at p < 0.05.

## Discussion

Glycolysis pathway has a different pattern in cancer when compared to normal cells because expression, structure and activity of some enzymes (e.g. pyruvate kinase) and some important master regulators (e.g. HIF-1, myc) are diverse [17,18]. The knowledge of glycolysis multiplicity is vital to comprehend the nature of the cancer cells in order to obliterate them because the best way to destroy an army is to identify all the parts of it and point out its weaker part as the Achilles’ heel.

It has been shown that the kinetic parameters of each enzyme in tissue are dependent on their environment. A few studies have been done which provide sufficient evidence of the alterations of kinetic properties of glycolytic enzymes in cancer cells. LDH is a key enzyme of aerobic glycolysis and attention has been paid to its kinetic parameters in cancer cell lines in recent years [19,20]. Those studies have come across an important problem; the tumor microenvironment has very heterogeneous oxygen pressure, pH and other metabolites [21] whereas the nature and importanceof the tumor microenvironment has been masked owing to the use of tissue culture conditions in which pH is normal without any fluctuation, also oxygen and nutrients are always in excess. This study was undertaken to compare the kinetic parameters of LDH in cancerous and normal tissues, with focus on the fact that enzyme kinetic parameters are dependent on their environment-involved enzyme. Our current study has shown the C-LDH in forward reaction has higher V_max_ compared to Normal N-LDH but the S_0.5_ of LDH between two tissues was not different. Elevation of C-LDH V_max_, with respect to its constant S_0.5_, can be due to increasein the total concentration of enzyme in tumor tissue. Higher V_max_ of C-LDH with respect to pyruvate and NADH showed significantly increased lactate and NAD^+^ production and increased pyruvate and NADH consumption. Three lines of evidence can explain our findings. Firstly, cancer cells require further levels of NAD^+^ for a continuing high glycolysis rate [4] and high activity of LDH in forward reaction supplies this requirement. Secondly, a high glycolysis rate in tumor cells increases the level of NADH. The increasing of NADH stimulates release of membrane bound LDH (A4) and more conversion of NADH to NAD^+^ with concomitant consumption of pyruvate A4 isoenzyme activity that catalyzes forward reaction will be increased in a metabolic environment containing low oxygen supply and ample pyruvate [22]. The two features exist in tumors, depressed supply of oxygen occurs in tumor microenvironment because blood vessels in tumors are often highly abnormal [23,24], also high glycolysis rate in cancer cells produces higher pyruvate level [25]. Our observations are consistent with the above clarification; the activity of LDH in forward reaction is higher in tumors than normal tissues. In addition, reducing the level of pyruvate by LDH in tumor tissues may assist cancer cells to maintain excessive growth and proliferation and may inhibit cancer cell death. To confirm this hypothesis Thangaraju et al. has shown that pyruvate prevents cell growth and proliferation by inhibition of histone deacetylase activity [26]. High LDH activity in forward reaction by altering pyruvate could eliminate the effect of pyruvate on cancer cell growth.

LDH in reverse reaction converts lactate to pyruvate with concomitant generation of NAD^+^ from NADH. Our results demonstrated that C-LDH had higher K_m_ for both lactate and NAD^+^ when compared with N-LDH. Contrary to our data, Debari et al. and Pizzuto et al. have shown that K_m_ lactate and K_m_ NAD^+^ were not different in PC3 and HEP G2 cell lines in relation to normal cells. The difference between our data and that reported by other researchers may be related to the culture environment that is a precise fit for cancer cell growth and proliferation, whereas the microenvironment of tumor tissue has various limitations that stimulates cancer cells to change their metabolism for persistent living and proliferation [21,27].

Higher K_m_ lactate and NAD^+^ means the C-LDH has a lower affinity for lactate and NAD^+^ to catalyze reverse reaction in tumor tissues, in other words, cancer cells resist converting lactate to pyruvate. This could be related to the key role of lactate in tumors. It has been shown that lactate contributes to the immune escape of cancer cells by inhibition of monocytes and dendritic cells differentiation and also by reduction of cytokine release from dendritic and cytotoxic T cells. In addition, the extrusion of high amount of lactate produced by aerobic glycolysis to the extracellular space could inhibit the lactate secretion from immune cells due to change in intra- to extracellular lactate balance. These changes suppress cellular immunity in tumor tissue [28]. Another possibility to explain our finding is the role of lactate in tumor metastasis and angiogenesis. Lactate indirectly enhances endothelial cell migration and angiogenesis by stimulating vascular endothelial growth factor (VEGF) production [29]. Lactate also increases the acidity of extracellular pH, a condition that stimulates invasion and metastasis of cancer cells [30]. The above mentioned traits of lactate make it very valuable for cancer cells, because according to Darwinian process, any traits that confer selective advantages to one population arepreserved by the population, therefore cancer cells should protect lactate because of its benefits in tumorigenesis. Low LDH affinity for lactate is one way of preserving lactate that is observedin the current study.

In addition, higher K_m_ lactate in C-LDH shows the C-LDH is less inhibited by high concentration of lactate and higher lactate levels could be tolerated. This feature of C-LDH may reflect one of the traits of the tumor microenvironment, which is a higher lactate level. Lactate level is significantly higher in tumors with metastatic spread in comparison to malignancies in patient without metastases [31,32]. Given the meaningful correlation between lactate concentrations in tumors with metastatic incidence, it can be concluded that the kinetic parameters of LDH, with respect to lactate, should be different in patients with metastases as one of the most effective factors on an enzyme kinetic is the characteristic of the environment that involves the enzyme.

The lower affinity of LDH for reverse reaction in tumors compared with normal tissues was emphasized by calculating the C-LDH and N-LDH efficiencies [33]. Our results showed that LDH efficiency was higher for lactate and NAD^+^ in normal tissues compared to tumors, which means that normal cells have more affinity to lactate in relation to tumor tissue which is consistent with the above mentioned explanations.

Our results demonstrated that E_a_ of reverse reaction was higher in tumors than that in normal tissues. This finding provides novel thermodynamic evidence about the inappropriate application of reverse reaction in tumor cells. In general, the higher values of K_m_ and E_a_ and lower enzyme efficiency for tumor LDH in reverse reaction showed cancer cells resist the conversion of lactate to pyruvate. It is important to note that substrate preference for reverse reaction by C-LDH and N-LDH may be due to their posttranslational modification during the tumorigenesis. Further investigation is needed to detect the posttranslational modification of LDH in cancer tissue and its effect on LDH structure and kinetic parameters.

## Conclusions

The results demonstrated that C-LDH has high affinity to produce lactate in forward reaction while it has low tendency to produce pyruvate in reverse reaction. Most studies have suggested inhibiting LDH activity in forward reaction [34] as one possible targeted therapy because the activity and expression of LDH in forward reaction is higher in cancer cells than normal cells. The current study showed for the first time that there is another possible approach to confront LDH effectiveness in cancer cells: if the affinity of LDH for lactate and NAD^+^can be increased, lactate is converted to pyruvate at higher rate and lactate dependent tumor growth and proliferation can be abolished. Further investigation is needed to confirm this hypothesis. In addition, the correlation between the kinetic parameters of LDH and the incidence of metastasis should be surveyed in more numbers of breast cancer patients. Finally, various enzymes kinetic parameters of different cancer cell lines should be investigated in culture conditions in which both oxygen and nutrients are restricted, similar to tumor microenvironment.

## Abbreviations

ATP: Adenosine three phosphate
LDH: Lactate dehydrogenase
NAD^+^: Oxidized form of nicotinamide adenine dinucleotide
NADH: Reduced form of nicotinamide adenine dinucleotide
EDTA: Ethylene diamine tetra acetic acid
EGTA: Ethylene glycol-bis(2-aminoethylether)-N,N,N’,N’- tetra cetic acid
PMSF: Phenyl methyl sulphonyl fluoride
K_m_: Michaelis-Menten constant
V_max_: Maximum velocity
E_a_: Activation energy
HIF: Hypoxia inducible factor
VEGF: Vascular endothelial growth factor
